# Enzyme additives influence bacterial communities of *Medicago sativa* silage as determined by Illumina sequencing

**DOI:** 10.1186/s13568-020-01158-5

**Published:** 2021-01-06

**Authors:** Zongfu Hu, Deying Ma, Huaxin Niu, Jie Chang, Jianhua Yu, Qing Tong, Shuguo Li

**Affiliations:** 1 College of Animal Science and Technology, Northeast Agricultural University, Harbin, People’s Republic of China; 2 College of Animal Science and Technology, Inner Mongolia University for Nationalities, Tongliao, People’s Republic of China

**Keywords:** Illumina sequencing, Bacterial community, Cellulase, ɑ-Galactosidase, *L. plantarum*, Alfalfa silage

## Abstract

The goal of the present study was to evaluate the effects of enzymes (cellulase combined with galactosidase) and their combination with *Lactobacillus plantarum* (LP) on bacterial diversity in alfalfa silages using high-throughput sequencing. Alfalfa forages were treated with or without cellulase + ɑ-galactosidase (CEGA), cellulase + LP (CELP), or ɑ-galactosidase + LP (GALP). After 56 days of ensiling, all treated silages exhibited improved fermentation quality, as reflected by decreased pH, ammonium-N and increased lactic acid levels compared to the control silage (*P* < 0.05). Enzymatic treatment improved nutrient value by increasing crude protein levels and decreasing neutral detergent fibre (NDF) levels (*P* < 0.05). Silage treatment significantly altered the bacterial community, as determined by PCoA (*P* < 0.05). Lactic acid bacteria (LAB) dominated the bacterial community of the treated silage after ensiling. The dominant bacteria changed from *Garciella*, *Enterococcus*, *Lactobacillus* and *Pediococcus* in the control silage to *Lactobacillus* and *Pediococcus* in the CEGA silage and *Lactobacillus* in the CELP and GALP silages. Collectively, these results suggest that treatment with both enzymes alone and in combination with inoculants greatly increased the abundance of LAB, with *Enterococcus*, *Lactobacillus* and *Pediococcus* observed in the silage treated with enzymes alone (CEGA) and *Lactobacillus* observed in the silage treated with a combination of enzymes and inoculants (CELP and GALP).

## Introduction

Alfalfa is a major forage for animal feed and is widely used worldwide. Due to the substantial dry matter (DM) loss that occurs during the hay-making process, ensiling is an efficient method for preserving the nutritive value of alfalfa (Oliveira et al. 2017; Muck et al. 2018). The efficient growth of LAB under anaerobic conditions can lead to the production of organic acids, inhibition of spoilage-associated bacterial and fungal growth, and the efficient preservation of silage nutrients, but a variety of epiphytic natural microbes affect fermentation (McDonald 1991).

In addition to bacterial inoculants, enzymes such as cellulase, hemicellulase, pectinase, and amylase (Tengerdy et al. 2010; Kozelov et al. 2008) are extensively used in the ensiling process (Muck et al. 2018; Dunière et al. 2013). The most commonly used enzymes are cellulases, which are widely recognized for their applications in forage preservation (Arriola et al. 2011; Contreras-Govea et al. 2011). These enzymes used for ensiling have the ability to degrade cell walls and release soluble sugars, which are essential substrates for LAB growth (Muck et al. 2018). The cleavage of β-(1,4)-linkages in cellulose by cellulase can release polysaccharides followed by glucose, decreasing the neutral detergent fibre (NDF) and acid detergent fibre (ADF) contents of silage (Nadeau et al. 2000). Previous studies have shown that treating silage with enzymes (cellulase, amylase, and pectinase) can increase lactate levels and decrease ammonia-N levels by 40% (Sheperd et al. 1995). A study by Selmer-Olsen et al. (1993) showed that cellulase/hemicellulase enzymes improve the silage quality of perennial ryegrass (*Lolium perenne*) and Italian ryegrass (*Lolium multiflorum*) and delay aerobic deterioration.

Currently, little research has been performed on the use of galactosidase as an additive in silage. Galactosidase, which is typically isolated from microbes (such as lactobacilli, fungi and yeasts), can act upon a variety of sugars, such as melibiose, raffinose and stachyose, by hydrolysing the (l-6) galactosidic bonds of these sugars to release glucose, which can be utilized by LAB (Mital et al. 1973; Garro et al. 1996). The glycosides raffinose, stachyose and saponin are present in alfalfa (Cunningham et al. 2003). Most glycosides are antinutritional factors (ANFs) for animals, which can inhibit the digestion and absorption of nutrients in animals and even cause poisoning. Saponins are the primary ANFs in alfalfa, and high levels of approximately 40 types of saponins, such as medicagenic acid, hederagenin, bayogenin, and soy saponins are present in alfalfa (Kiełbasa et al. 2019). The common sugars that make up saponins include glucose, galactose, rhamnose, arabinose, xylose and other pentose sugars (Pecetti et al. 2010; Tava and Odoardi 1996; Rafińska et al. 2017). The ability to hydrolyse ɑ-galactosidase aids in the removal of ANFs. Furthermore, the hydrolysis of this saponin to monose, such as glucose or pentose, can promote the growth of LAB and improve the fermentation quality of alfalfa silage.

Bacterial community composition is important for the fermentation quality of silage. To date, several studies have investigated the bacterial community of silage treated with inoculants, such as, *Lactobacillus plantarum*, and *Lactobacillus buchneri* (Drouin et al. 2019; Zheng et al. 2017; Guo et al. 2018; Yang et al. 2019). However, the effects of enzyme treatment and further treatment with enzyme + inoculants on silage microbiota have not been investigated and remain unknown. Therefore, the objective of the present study was to investigate the differences in the bacterial communities in alfalfa silage treated with enzymes, inoculants and their combinations. Using the Illumina MiSeq platform, we characterized the bacterial community of alfalfa silage inoculated without and with cellulase plus ɑ-galactosidase (CEGA), cellulase plus LP (CELP), and ɑ-galactosidase plus LP (GALP).

## Materials and methods

### Sample preparation and collection

Alfalfa (50% bloom stage) was harvested at the second cut from fields located at the University of Inner Mongolia for Nationalities (E122°15′, N43°38′), Inner Mongolia, on June 22, 2017. The alfalfa was wilted to obtain a DM content of approximately 37% in a ventilated room and then chopped to a length of 10 mm with a forage cutter. The forage was treated as follows: (1) control with no additives; (2) cellulase (20,000 IU/g, SD-124, Challenge Co., Ltd., Beijing, China) plus ɑ-galactosidase (15,000 IU, SD-124, Challenge Co., Ltd., Beijing, China) (CEGA) at a dose of 5 g/kg fresh forage for each enzyme; (3) cellulase at a dose of 5 g/kg forage combined with 1 × 10^7^ cfu/g FM of freshly cultured *L. plantarum* (BNCC337987, Bnbio Co., Ltd., Beijing, China) (CELP); and 4) ɑ-galactosidase at a dose of 5 g/kg forage, combined with 1 × 10^7^ cfu/g FM of *L. plantarum* (GALP). During the preparation of silage, the additives were applied to the forage as a solution (20 ml/kg FM), and untreated silage was sprayed with equal amounts of sterile water. Five hundred grams of the forage was sealed in polyethylene silo bags using a vacuum sealer (BH 950, Matsushita, Tokyo, Japan) to remove the air. Twelve vacuum-bag mini-silos were prepared, with 3 repetitions performed for each treatment. Ensiling was performed at room temperature (26 °C) for 56 days.

### Chemical analyses and microbial enumeration

The fresh forage (d 0 sample) and all silage samples were analysed for their chemical characteristics. The DM levels of the samples were determined in a forced-air oven at 60 °C for 48 h. The ammonia-N (NH_3_-N) content was measured as described by Zahiroddini et al. (2004). The water-soluble carbohydrate (WSC) content was measured according to Owens et al. (1999). To obtain the silage extract, 20 g of each sample was mixed with 180 mL of deionized water. After overnight incubation at 4 °C, the sample was shaken for 2 min and then filtered through coarse (20–25 μm particle retention) filter paper. Silage pH and the levels of ammonia-N and organic acids (lactic, acetic, propionic, and butyric acids) were measured using the water extracts of the silages (Kung et al. 2003). Organic acid levels were measured by HPLC on an Agilent 1100 system (Agilent, Palo Alto, CA, USA) equipped with a UV detector (210 nm) and a column (ICSep COREGEL-87H). The mobile phase was 0.005 M H_2_SO_4_ at a flow rate of 0.6 mL/min at 55 °C (Ni et al. 2017). The NDF and ADF levels were measured as described by van Soest et al. (1991). The crude protein (CP) content was measured using the Kjeldahl N method and calculated as Kjeldahl N × 6.25 (AOAC 2000). LAB was enumerated by plate counting on de Man, Rogosa and Sharpe (MRS) agar.

### DNA extraction and Illumina sequencing

Microbial DNA was extracted from raw silage samples using a FastDNA SPIN Soil Kit (MP Biomedicals, Santa Ana, CA, USA). In brief, silage (approximately 0.3–0.4 g wet weight for each sample) was first added to a 2 ml lysing matrix tube added with homogenizing reagent provided by kit, then employed a series of lysing, homogenizing, and DNA purification and elution procedure following the manufacturer’s instructions. DNA quality was checked by 1% agarose gel electrophoresis and spectrophotometry (NanoDrop 2000, Thermo Scientific, Waltham, MA, USA; 260/280 nm optical density ratios). PCR was used to amplify the variable (V3-V4) region of the 16S rRNA genes using 12-bp barcoded primers (forward 338F: 5′-ACTCCTACGGGAGGCAGCA-3′; reverse 806R: 5′-GGACTACHVGGGTWTCTAAT-3′). PCR products were extracted from a 2% agarose gel and purified with a AxyPrep DNA Gel Extraction Kit (Axygen Biosciences, Union City, CA, USA). Finally, the cloned libraries were pooled in equimolar amounts and sequenced on the Illumina MiSeq PE300 platform (Illumina Corporation, San Diego, CA, USA) at Shanghai Majorbio Bio-Pharm Technology Co., Ltd. (Shanghai, China).

The sequence data for all samples were deposited in the NCBI Sequence Read Archive (SRA) under BioProject number PRJNA522947.

### Bioinformatics analysis

After sequencing, the fastq files for each sample were generated. Paired-end sequences were merged by their overlapping regions with overlaps of > 10 bp using Trimmomatic (Bolger et al. 2014). QIIME (Caporaso et al. 2010) was used to perform quality control. Usearch (version 7.1) (Edgar et al. 2011) was used to identify operational taxonomic units (OTUs) based on 97% sequence identity and check the sequence quality. The representative sequences obtained for each OTU were compared with Silva 132 (http://www.arb-silva.de) to obtain taxonomic information with RDP Classifier (version 2.2, http://sourceforge.net/projects/rdp-classifier/).

ɑ-Diversity indices (Shannon, Simpson, Chao, and Ace) were determined with mothur software (version v.1.30.1) (Schloss et al. 2009). Non-metric multidimensional scaling (NMDS) analysis was performed to evaluate the β-diversity distance matrix, and Adonis analysis was performed to test the reliability of the NMDS clusters using QIIME. Redundancy analysis (RDA) was conducted in the R vegan package using Monte Carlo permutation (999 repetitions).

### Statistical analysis

All data from chemical determinations, and microbial counts (transformed to log10) were evaluated by one-way analysis of variance using SPSS 19.0 (SPSS, Chicago, IL, USA), with the different treatments of alfalfa silages were regarded as the fixed effect. Using the calculated means, the data were further compared with Tukey’s test at the 5% significance level. All data are presented as the mean ± standard error of triplicate groups.

## Results

### Chemical composition

The DM content, pH, WSC, ammonia, crude protein, NDF, and ADF values of alfalfa forage prior to ensiling were 36.92%, 6.13, 69.23 g/kg DM, 4.58 g/kg DM, 24.62 g/kg DM, 47.64 g/kg DM, and 36.73 g/kg DM, respectively. The number of detected LAB was 6.37 log cfu/g FM (Table 1).

**Table 1:** Chemical composition of alfalfa forage prior to ensiling

DM	pH	WSC	NH3-N	Protein	NDF	ADF	Fat	LAB
36.9 ± 0.73	6.1 ± 0.04	69.2 ± 1.12	4.6 ± 0.07	24.6 ± 0.23	47.6 ± 0.67	36.7 ± 0.44	4.8 ± 0.04	6.4 ± 0.08

Compared to alfalfa forage prior to ensiling, ensiling decreased the DM, protein, pH, and WSC values and increased the number of LAB (Tables 1, 2). After ensiling, the DM content of the treated silages was higher than that of the control silage, and the DM content of the CEGA and GALP silages was higher than that of the CELP silage (*P* < 0.05). The pH values of all the treated silages were lower than that of the untreated silage, while the pH of the CELP silage was lower than that of the CEGA and GALP silages (*P* < 0.05). The ammonia levels in the treated silages were lower than those in the control silage, while those in the CEGA silage were lower than those in the CELP and GALP silages (*P* < 0.05). The LAB counts were higher in treated silages than in the control silage (*P* < 0.05), while no differences were observed among the treated silages (*P* > 0.05) (Table 2).

**Table 2:** Fermentation characteristic and microbial counts of the alfalfa silages (g/kg of DM except as noted)

	Control	CEGA	CELP	GALP
DM g/100 g	33.5 ± 0.31^c^	35.9 ± 0.41^a^	35.0 ± 0.20^b^	36.2 ± 0.32^a^
pH	5.2 ± 0.18^a^	4.8 ± 0.04^bc^	4.7 ± 0.07^c^	4.9 ± 0.09^b^
WSC g kgDM-1	13.0 ± 0.73^c^	14.9 ± 1.06^bc^	13.5 ± 0.93^a^	13.8 ± 0.62^b^
*Fermentation products*				
Ammonia (g kgDM-1)	7.8 ± 0.41^a^	4.6 ± 0.29^d^	5.3 ± 0.61^b^	5.0 ± 0.25^c^
Lactic acid (g kgDM-1)	30.7 ± 1.17^d^	36.9 ± 2.36^c^	51.2 ± 2.41^a^	45.5 ± 2.04^b^
Acetic acid (g kgDM-1)	12.6 ± 1.48^c^	38.3 ± 1.94^a^	17.5 ± 0.98^b^	18.4 ± 1.09^b^
propionic acid (g kgDM-1)	1.8 ± 0.07^a^	0.2 ± 0.01^b^	0.2 ± 0.06^b^	0.3 ± 0.01^b^
Butyric acid (g kgDM-1)	3.1 ± 0.23^a^	0.0 ± 0.00^b^	0.0 ± 0.02^b^	0.1 ± 0.04^b^
LAB(log cfu g^−1^ FM)	7.5 ± 0.16^b^	8.3 ± 0.14^a^	8.5 ± 0.08^a^	8.5 ± 0.12^a^
*Nutrient composition*				
Crude protein	21.7 ± 0.09^c^	22.6 ± 0.25^a^	23.5 ± 0.59^a^	23.3 ± 0.02^b^
NDF	43.1 ± 1.70^a^	41.7 ± 1.86^b^	40.2 ± 2.05^c^	42.6 ± 1.43^b^
ADF	34.9 ± 0.56^a^	32.3 ± 0.98^b^	31.3 ± 1.59^b^	33.3 ± 0.36^ab^
Crude Fatty	4.3 ± 0.05^a^	4.3 ± 0.03^a^	3.7 ± 0.05^b^	3.7 ± 0.04^b^

*CON* untreated silage, *CEGA* silage treated with cellulase plus ɑ-galactosidase, *CELP* silage treated with cellulase plus *L. plantarum*, *GALP* silage treated with ɑ-galactosidase plus *L. plantarum*

^a, b, c^Means with different superscripts in the same row differ (*P* < 0.05)

After ensiling, the treated silages showed higher lactic acid levels than the untreated silage (*P* < 0.05), particularly for the CELP silage, which was higher than that observed in the CEGA and GALP silages. The treated silages (CEGA and GALP) exhibited increased acetic acid levels compared to the control silage, while the CEGA silage showed higher acetic acid contents than the CELP and GALP silages (*P* < 0.05). No differences in the levels of propionic and butyric acids were observed among the treated silages (*P* > 0.05), while the levels of these acids were lower in the treated silages than in the untreated silage (*P* < 0.05).

The treated silages (CEGA, CELP, and GALP) exhibited lower NDF contents than the control silages (*P* < 0.05), where the NDF level was lower in the CELP silage than in the CEGA and GALP silages (*P* < 0.05). All treated silages had lower ADF contents than the control silage (*P* < 0.05), with no significant differences observed between the treated silages (*P* > 0.05). The crude protein contents of the treated silages were higher than that of the control silage, and the CEGA silage showed lower levels of crude protein than the CELP and GALP silages (*P* < 0.05). No significant differences in crude protein levels were observed between the CELP and GALP silages (*P* > 0.05). The fat content in the control and CEGA silages was higher than that in the CELP and GALP silages (*P* < 0.05) (Table 2).

### Sequence analysis of bacterial communities in alfalfa silages

Based on Illumina sequencing of the V3-V4 region of the 16S rRNA gene for bacteria in silage, an average of 34,095 ± 3107 sequences were obtained from each sample, with an average length of 448 ± 4 bp per sequence. All sequences were sub-sampled to a size of 29,271 to limit sampling error. Finally, 69 OTUs were obtained that were assigned to 59 species, 45 genera, 30 families, 19 orders, 13 classes and 7 phyla. The tendency of the rarefaction curves to plateau indicates that sufficient sequencing depth of the silage bacterial communities was achieved (Additional file 1: Figure S1). With respect to the ɑ-diversity indices, GALP showed higher Chao and Ace indices compared to that of the control silage (*P* < 0.05) (Table 3).

**Table 3:** Statistics of high-throughput sequencing data and the bacterial community diversity of alfalfa silage

Treatments	Sobs	Shannon	Simpson	Chao	Ace
Control	32.0 ± 9.54	1.8 ± 0.54	0.3 ± 0.17	33.0 ± 8.66^b^	35.3 ± 6.20^b^
CEGA	29.0 ± 3.46	1.5 ± 0.25	0.3 ± 0.08	34.0 ± 9.00^ab^	34.4 ± 9.20^ab^
CELP	34.7 ± 4.04	1.5 ± 0.03	0.3 ± 0.01	38.8 ± 6.33^ab^	46.0 ± 15.12^ab^
GALP	39.0 ± 7.00	1.5 ± 0.10	0.3 ± 0.02	42.6 ± 5.65^a^	50.0 ± 6.54^a^

*CON* untreated silage, *CEGA* silage treated with cellulase plus ɑ-galactosidase, *CELP* silage treated with cellulase plus *L. plantarum*, *GALP* silage treated with ɑ-galactosidase plus *L. plantarum*

^a, b, c^Means with different superscripts in the same column differ (*P* < 0.05)

### Clusters of bacterial communities between alfalfa silages

**Fig. 1:**
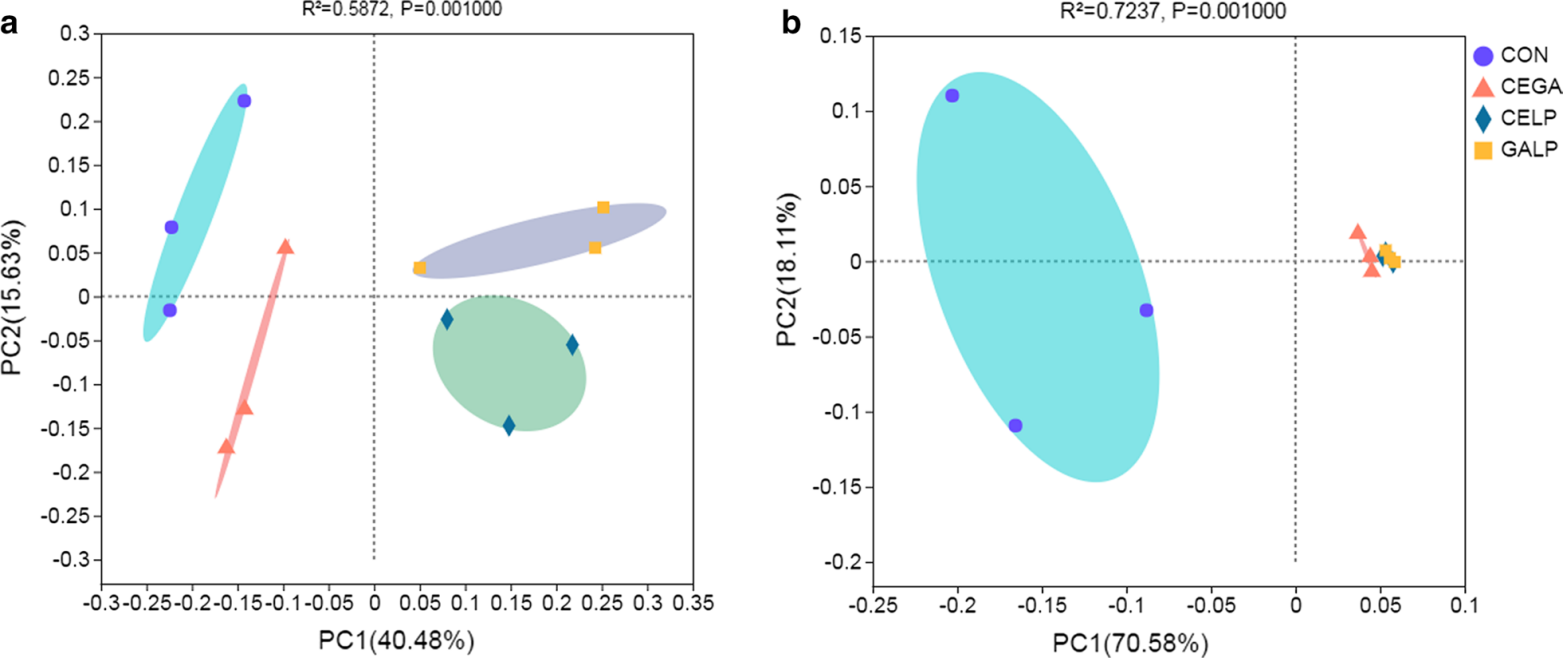
Principal coordinate analysis (PCoA) of the bacterial communities based on unweighted (**a**) and weighted UniFrac (**b**) at the OTU level. *CON* untreated silage, *CEGA* cellulase plus ɑ-galactosidase, *CELP* cellulase plus *L. plantarum*, *GALP* ɑ-galactosidase plus *L. plantarum*

PCoA based on weighted UniFrac distances revealed distinct clusters between the control and additive-treated silages (Fig. 1), including for the non-additive-treated silage, enzyme-treated silage (CEGA), and silage treated with enzyme in combination with inoculant (CELP and GALP). Further analysis using the Adonis test showed that the clusters were reliable (R^2^ = 0.72, *P* = 0.001). Venn diagram analysis showed that significant overlap occurs between the control silage and CEGA silages, with 32 OTUs shared by these two groups. The CELP and GALP silages were similar, with 38 OTUs shared by these two groups (Additional file 2: Figure S2). These results clearly indicate that the inoculants affected the bacterial community of the alfalfa silage.

### Bacterial composition of alfalfa silage

**Fig. 2:**
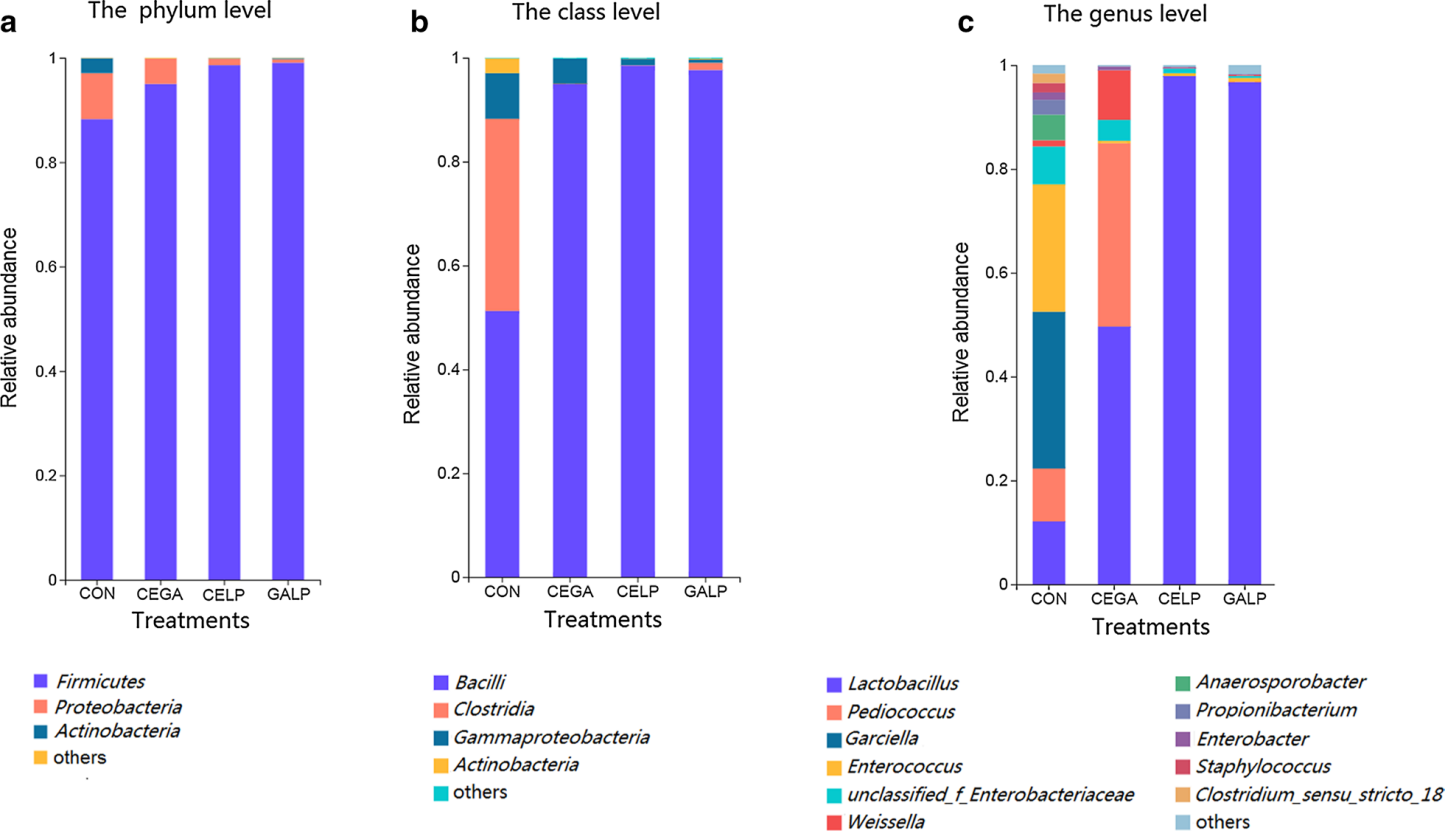
Bacterial composition of alfalfa silage with different treatments (Control, CEGA, CELP, and GALP) at the phylum (**a**), class (**b**) and genus levels (**c**). Phyla, classes and genera with less than 1% overall relative abundance were summed and presented as “others”. *CON* untreated silage, *CEGA* cellulase plus ɑ-galactosidase, *CELP* cellulase plus *L. plantarum*, *GALP* ɑ-galactosidase plus *L. plantarum*

At the phylum level, *Firmicutes* dominated the bacterial communities of the control silage, with an observed abundance of 88.28%, followed by *Proteobacteria* and *Actinobacteria*, with observed abundances of 8.79 and 2.86%, respectively. *Firmicutes* dominated the bacterial communities of all treated silages, with observed abundances of 95.01, 98.57, and 99.07% observed in the CEGA, CELP and GALP silages, respectively. *Proteobacteria* was also observed in all treated silages at a low abundance (Fig. 2a).

At the class level, *Bacilli* and *Clostridia* were predominant in the control silage, with observed abundances of 51.26 and 37.01% respectively, while *Bacilli* dominated the bacterial communities of all treated silages, with abundances of 94.98, 98.53, and 97.66% observed in the CEGA, CELP and GALP silages, respectively. *Gammaproteobacteria* were detected in all silages, with observed abundances of 8.79, 4.82, 1.22, and 0.62% in the control, CEGA, CELP, and GALP silages, respectively. *Actinobacteria* was only detected in the control silage, with an observed abundance of 2.87% (Fig. 2b). The abundance of *Bacilli* in the control silage was lower than that in all the treated silages (*P* < 0.05). The abundances of *Clostridia* and *Actinobacteria* in the control silage were higher than those in all the treated silages (*P* < 0.05) (Additional file 3: Figure S3).

Bacterial composition changes in the silage were also observed at the genus (Fig. 2c, Additional file 4: Figure S4) and OTU (Additional file 5: Figure S5) levels. Eleven genera exhibited a relative abundance > 1% in the control silage. Among these genera, *Garciella*, *Enterococcus*, *Lactobacillus* and *Pediococcus* showed abundances of 30.21, 24.54, 12.11, and 10.18%, respectively. In addition, *Clostridium_sensu_stricto*_18 (1.82%), *Staphylococcus* (1.81%), *Propionibacterium* (2.84%), and *Anaerosporobacter* (4.93%) also exhibited abundances above 1%.

**Fig. 3:**
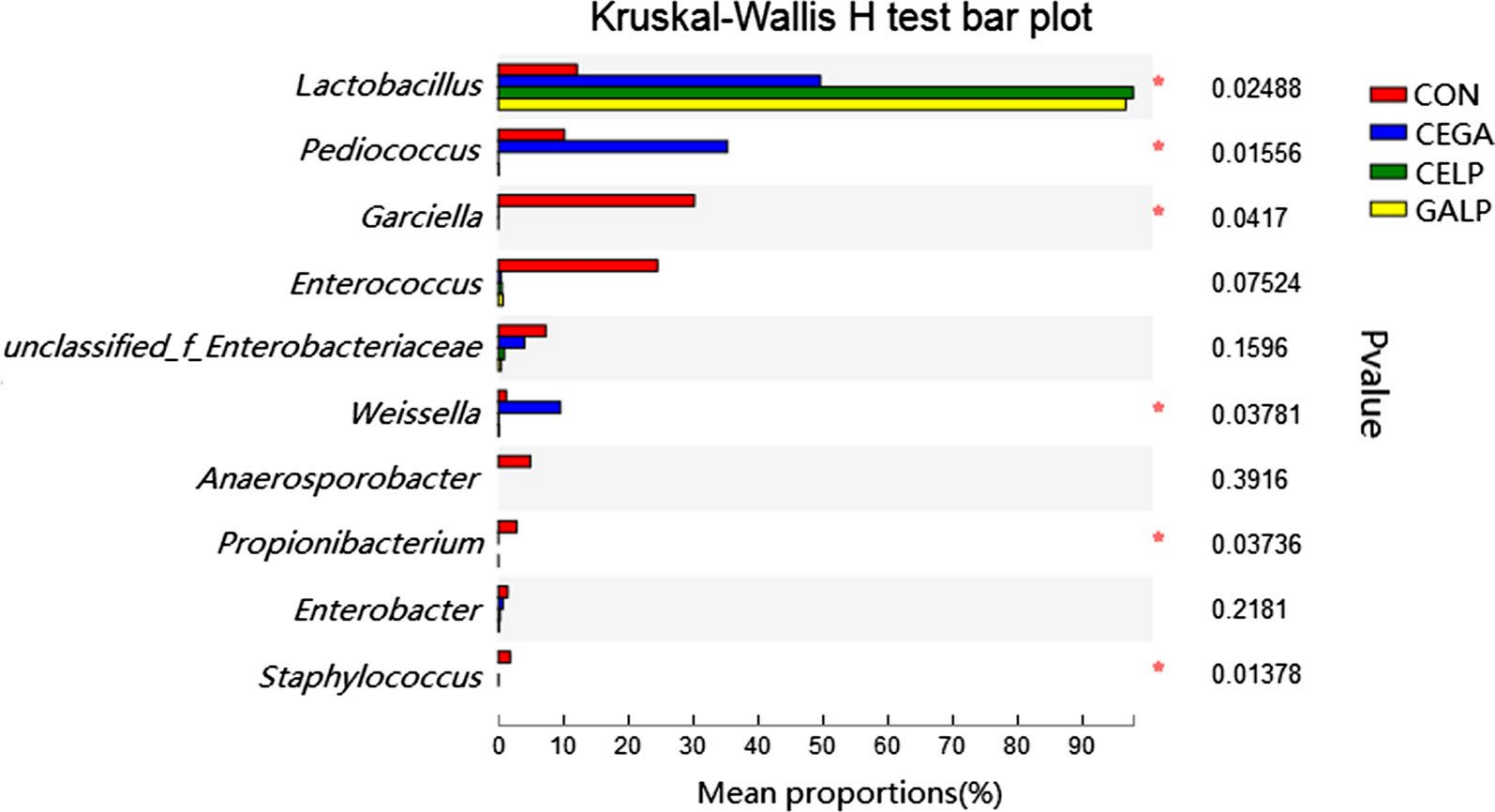
Kruskal–Wallis H test of the bacterial community at the genus level. The top 10 genera are shown in the figure. *CON* untreated silage, *CEGA* cellulase plus ɑ-galactosidase, *CELP* cellulase plus *L. plantarum*, *GALP* ɑ-galactosidase plus *L. plantarum*

In the CEGA silage, compared to the control silage, *Lactobacillus* and *Pediococcus* exhibited increased abundances of 49.64 and 35.31%, respectively (*P* < 0.05). *Weissella* was also present in the CEGA silage at a relatively high abundance (9.56%) compared to that in the control silage (*P* < 0.05) (Figs. 2c, 3).

The other three silages showed similar bacterial compositions, with *Lactobacillus* being the dominant genus at an abundances of 97.88 and 96.74% in the CELP and GALP silages, respectively. In addition, no genera were above a 1% abundance in these silages (Fig. 2c, Additional file 4: Figure S4).

Significant differences in the abundances of many bacterial taxa were observed between groups, as analysed by the Kruskal–Wallis H test (*P* < 0.05) (Fig. 3). The abundance of *Lactobacillus* in the control silage was lower than that in all treated silages (CEGA, CELP, and GALP) (*P* < 0.05). Furthermore, the abundance of *Lactobacillus* in the CELP and GALP silages was higher than that in the CEGA silage (*P* < 0.05). The abundance of *Garciella* was higher in the control silage than in all the treated silages (*P* < 0.05), while that of *Pediococcus* in the CEGA silage was higher than that in the control, CELP and CEGA silages (*P* < 0.05). In addition, the abundance of *Propionibacterium* in the control silage was higher than in the CEGA, CELP, and GALP silages (*P* < 0.05).

### Relationship between fermentation products and bacterial communities

**Fig. 4:**
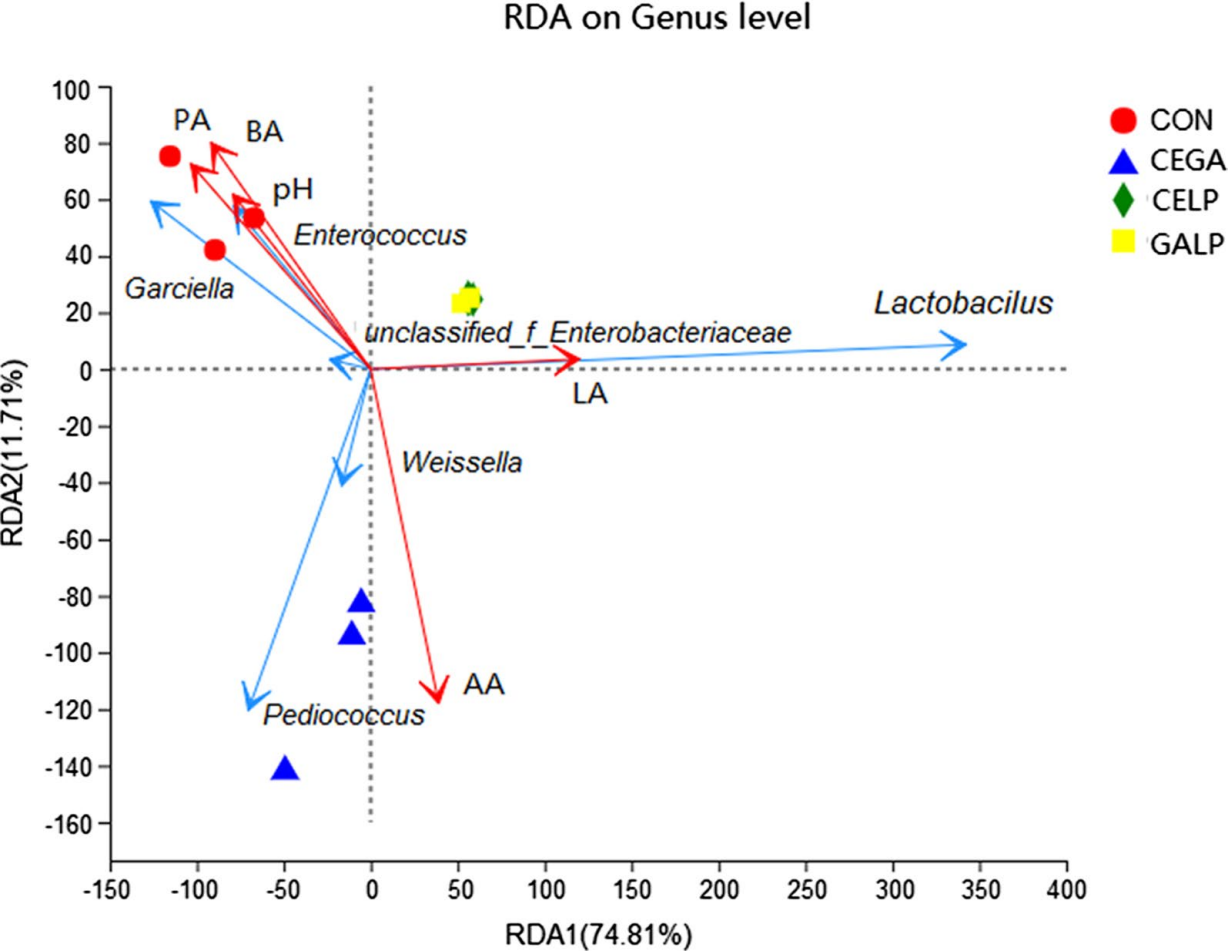
Correlation analyses between microorganisms and fermentation products in alfalfa silage. *LA* lactic acid, *AA* acetic acid, *PA* propionic acid, *BA* butyric acid, *CON* untreated silage, *CEGA* cellulase plus ɑ-galactosidase, *CELP* cellulase plus *L. plantarum*, *GALP* ɑ-galactosidase plus *L. plantarum*

RDA was performed to evaluate the effects of bacterial composition on the fermentation products in alfalfa silages (Fig. 4). Mantel tests on the models were performed with a minimum of 999 permutations, and the multivariate regression was significant (*R* = 0.49, *P* = 0.005), indicating a correlation between the bacterial composition and fermentation products. As demonstrated by RDA results, *Lactobacillus* was associated with the lactic acid content in the CELP and GALP silages, whereas *Caeciella* and *Enterococcus* were associated with the pH value and propionic and butyric acids contents in the control silage. In addition, *Pediococcus* and *Weissella* were associated with the acetic acid content in the CEGA silage.

## Discussion

### Effects of enzyme and enzyme + inoculant treatments on silage fermentation quality

The treatment of silage with enzymes (cellulase combined with galactosidase) alone or in combination with *L. plantarum* affected the LAB cfu of silage after 56 days. The increased organic acid levels in the alfalfa silages suggested that the enzyme treatments were markedly beneficial, especially enzyme plus inoculant treatments (cellulase plus *L. plantarum* and galactosidase plus *L. plantarum*) (Nadeau et al. 2000). Concurrently, enzyme-treated (CEGA) silage exhibited increased acetic acid concentrations. Consistent with our results, Kung et al. (1991) observed increased acetic acid concentrations in cellulase plus pectinase-treated alfalfa silage, whereas a decreased acetic acid concentration was observed in alfalfa silage treated with cellulase (Nadeau et al. 2000). The decreased acetic acid levels indicate that more homolactic than heterolactic fermentation occurred in silage. The lactic acid concentration in the silage treated with inoculants + enzyme were higher than those in the silage treated with enzyme only, indicating that combined enzyme and inoculants treatment may be more beneficial than the enzyme alone treatment. These results indicate that the addition of inoculants amplified the effect of the enzyme and led to a more rapid fermentation with lactic acid production. Overall, the treatment of alfalfa silage with enzymes or enzymes combined with inoculants clearly improved the ensiling characteristics, decreasing the pH, ammonia levels, and butyric acid levels and increasing the DM and lactic acid levels. Consistent with our results, previous studies showed that fibrolytic enzyme treatment resulted in increased lactic acid levels and decreased pH, butyric acid and ammonia levels compared with the control in the silages of various plants, such as lucerne, barley and orchardgrass (Dehghani et al. 2012; Zahiroddini et al. 2004; Nadeau et al. 2000). Notably, the addition of enzymes (CEGA, CELP, and GALP) as a whole increased protein levels, and decreased the NDF and ADF levels. On the one hand, the reduction of NDF and ADF levels increases the proportion of protein; on the other hand, the soluble sugars released by cellulose hydrolysis increase the number of LAB, which inhibits the growth of spoilage microorganisms and protein degradation by spoilage microorganisms. This finding indicated the degradation of the plant cell walls by fibrolytic enzymes (Muck et al. 2018), which is consistent with the results of a study of Lynch et al. (2014), who showed that fibrolytic enzymes decreased both NDF and ADF concentrations in alfalfa silage. Similarly, van Vuuren et al. (1989) observed that cell wall degrading enzymes decreased the NDF and ADF levels of grass silage. In other studies, the NDF, ADF and protein levels did not considerably change upon treatment with inoculants or inoculants plus enzyme (Fredeen et al. 1991; Kozelov et al. 2008), possibly due to a high DM content. Tengerdy et al. (1991) used cell wall degrading enzymes or a combination of these enzymes with LAB to ensile fresh cut and wilted alfalfa and showed that a cocktail of cellulase, hemicellulase and pectinase enzymes with a lactic acid bacteria inoculum containing *Pediococcus*, *Lactobacillus* and *Streptococcus* spp*.* is more effective in producing lactic acids and the enzyme treatments were more beneficial in terms of improving ensiling quality in fresh rather than wilted alfalfa, perhaps indicating that the high moisture conditions are favourable for enzyme hydrolysis.

### Bacterial communities in alfalfa silage

Regarding the microbial diversity of silage treated with enzymes, relevant research is lacking. Therefore, the bacterial communities of alfalfa silage treated with enzymes and enzymes + inoculants were evaluated in the present study. Compared to untreated silage, the enzyme and combination of enzyme and inoculant treatments increased the total abundance of LAB in silage. In the untreated silage, several LAB, including *Enterococcus*, *Pediococcus*, *Lactobacillus*, and *Weissella* species accounted for 46.83% of the total abundance at the genus level. The enzymatic treatment of silage apparently increased the abundance of LAB to 94.54% at the genus level, including *Lactobacillus* (49.64%), *Pediococcus* (35.31%), and *Weissella* (9.56%), which was approximately double that of the control silage. As discussed above, fibrolytic enzymes in silage can degrade fibre to glucose, which can be used by LAB, as the results showed. The increase of glucose in silage due to treatment with fibrolytic enzymes was showed in lucerne, corn and Italian ryegrass (Dehghani et al. 2012; Shepherd et al. 1995; Selmer-Olsen et al. 1993; Shepherd and Kung 1996).

Lactic acid bacteria (LAB) are important for good quality silage owing to their production of organic acids, primarily lactic acids. As shown in the present study, the high abundance of LAB led to the sufficient production of organic acids and greatly reduced the silage pH (Table 1). Lactic acid bacteria (LAB) can also produce antimicrobial substances, such as bacteriocins and reuterin, which inhibit other spoilage bacteria (Klaenhammer 1988). In the present study, compared to the control silage, the disappearance of the class *Clostridia* in the both enzyme and inoculants-treated silages may be a result of the high level of LAB, a mechanism that was confirmed by the observed of bacteriocin production (Flythe and Russell 2004; Marcinakova and Laukova 2004).

*Lactobacillus* was present at a high abundance in the enzyme-treated silage and exhibited absolute dominance in silages treated with enzyme plus inoculant (CELP and GALP). The dominance of *Lactobacillus*, leading to the elimination of native bacteria and decreased diversity of the bacterial community in silage treated with inoculants, was also observed in other studies (Li and Nishino 2011; Parvin et al. 2010; Guo et al. 2018). *Lactobacillus* is a complex bacterial genus comprising both homofermentative and heterofermentative LAB. Depending on the species, homofermentative LAB can produce lactic acid, while heterofermentative LAB can produce both lactic and acetic acids (Muck et al. 2018). Corresponding to the absolute dominance of *Lactobacillus*, the significantly higher lactic acid content in the CELP and GALP silages than that in the control and enzyme-treated silages indicates that the increase in *Lactobacillus* is more effective for the production of lactic acid than other LAB species.

The treatment of cellulose + *L. plantarum* and ɑ-galactosidase + *L. plantarum* led to the absolute domination by *Lactobacillus* in the alfalfa silage bacterial community, which was similar to the *L. plantarum*-treated alfalfa silage observed in previous studies, which also showed the dominance of *Lactobacillus* (Bao et al. 2016; Zheng et al. 2017; Yang et al. 2019). These results indicate the active growth of *Lactobacillus* in the *L. plantarum*-treated alfalfa silage, which may not be affected by enzyme treatment to some extent.

Bacteria of the genus *Bacillus* as well as *Enterobacteriaceae* species, such as those of the genus *Enterobacter*, were also clearly present in the untreated silage and exhibited decreased abundances in the treated silages. These bacteria can produce 2,3-butanediol by expending sugars needed by LAB (McDonald 1991), which may inhibit the growth of LAB in untreated control silage. The same phenomenon was observed for *Anaerosporobacter* species, which also ferment various carbohydrates to produce acetic and formic acids (Jeong et al. 2007). *Aeriscardovia* was present in silages treated with inoculants plus enzyme. *Aeriscardovia*, which was first isolated from a porcine caecum (Simpson et al. 2004), belongs to the family *Bifidobacteriaceae*, members of which can ferment a variety of carbohydrates to organic acids, such as acetic and lactic acids (Biavati and Mattarelli 2006).

### Correlation of bacterial community with fermentation quality

RDA results showed that some bacterial genera were correlated with the fermentation indices, such as the positive correlation of *Lactobacillus* with lactic acids, *Caeciella* and *Enterococcus* with pH, propionic acid and butyric acid, and *Pediococcus* with acetic acid. *Lactobacillus* is the genus that is typically present in the highest abundance in silages treated with LAB, and it contributes the most to the production of lactic acids and the decline in pH during fermentation (Li et al. 2019; Zheng et al. 2017; Ni et al. 2017; Cai et al. 2020; Keshri et al. 2018). Furthermore, the correlation between *Lactobacillus* and lactic acids has been in other studies (Yang et al. 2019; Su et al. 2019).

*Pediococcus*, *Enterococcus* and *Weissella* were predominant in the enzyme-treated silage, which was consistent with the high level of acetic acid observed in this silage. As a genus of facultative heterofermentative LAB, *Pediococcus* can produce lactic acid by fermentation of hexoses and can also produce lactic and acetic acids via the fermentation of pentose (Muck et al. 2018). *Enterococcus* is also a genus of facultative heterofermentative bacteria. The occurrence of these two bacteria may explain the high acetic acid levels observed in the enzyme-treated (CEGA) silages. *Weissella*, which was present at a specific abundance in enzyme added silage (CEGA) silage, is a heterofermentative LAB that also contributed to the production of lactic and acetic acids. Although *Enterococcus* was present in the control silage, the low acetic acid levels in the control silage may be caused by the low abundance of LAB as a whole and the presence of *Garciella*, which can utilize organic acids as a fermentation substrate (Miranda-Tello et al. 2003).

The ammonia, propionic acid, and butyric acid levels in the control silage were distinctly higher than those in the treated silage. These substances can be produced by *Garciella* species, which were predominant in the untreated silage and absent in the treated silages. *Garciella* belongs to Clostridia, and was previously detected in alfalfa silage using a specific primer set to amplify the 16S rRNA gene from Clostridia species (Zheng et al. 2017). In addition to fermenting sugars to produce lactic, acetic, and butyric acids, *Garciella* species are capable of utilizing organic acids, such as lactic acid to produce butyric acid, and can also produce hydrogen sulphide by reducing thiosulphate and ammonium by reducing nitrate (Miranda-Tello et al. 2003). Both butyric acid and ammonium are not considered to be desirable matter in silage.

Taken together, these findings indicate that the enzyme and enzyme plus LP treatments improved the silage fermentation quality, which decreased pH and ammonia levels and increased lactic acid levels. The nutritional value was also improved by preserving more DM and crude protein and decreasing the NDF level. The bacterial communities of the alfalfa silages exhibited changes as a result of the enzyme treatment, as revealed by high-throughput sequencing. Enzymatic treatment of alfalfa resulted in the predominance of several LAB genera in the silage, including *Lactobacillus*, *Pediococcus*, *Enterococcus* and *Weissella*, but the enzyme combined with LP treatment greatly increased the abundance of *Lactobacillus* in the alfalfa silage. The relationship between the bacterial community of the silage and ensiling characteristics was also confirmed in the present study. Therefore, the results of the present study support the treatment of silage with enzymes combined with LP, which showed a high fermentation quality and dominance of *Lactobacillus* in the alfalfa silage.

## Supplementary Information

**Additional file 1: Figure S1.** Rarefaction curves showing the OUT diversity in alfalfa silages. *CON* untreated silage, EN silages treated ith cellulose plus α-galactosidase, *LP* silage treated with *L. plantarum*, ENLP silage treated with cellulase plus α-galactosidase plus *L. plantarum*.

**Additional file 2: Figure S2.** Venn showing the shared and unique OUT of bacterial communities in different treatments of alfalfa silage. *CON* untreated silage, *CEGA* silages treated with cellulase plus α-galactosidase, *CELP* silages treated with cellulase plus *L. plantarum*, GALP silages treated with α-galactosidase plus *L. plantarum*.

**Additional file 3: Figure S3.** Kruskal-Wallis H test of bacterial community at class level. The top 5 classes were accounted in the figure. *CON* untreated silage, *CEGA* silages treated with cellulase plus α-galactosidase, *CELP* silages treated with cellulase plus *L. plantarum*, *GALP* silages treated with α-galactosidase plus *L. plantarum*.

**Additional file 4: Figure S4.** Community barplot analysis showing bacterial composition of the alfalfa silage at genus level. *CON* untreated silage, *CEGA* silages treated with cellulase plus α-galactosidase, *CELP* silages treated with cellulase plus *L. plantarum*, *GALP* silages treated with α-galactosidase plus *L. plantarum*.

**Additional file 5: Figure S5.** Community barplot analysis showing bacterial composition of the alfalfa silage at OUT level. *CON* untreated silage, *CEGA* silages treated with cellulase plus α-galactosidase, *CELP* silages treated with cellulase plus *L. plantarum*, *GALP* silages treated with α-galactosidase plus *L. plantarum*.

## Data Availability

All data generated or analysed during this study are included in this published article and its additional files. Sequence datasets for all the samples were deposited in the NCBI Database under accession number PRJNA522947.
