# Supplementary figures and images for: Enzyme additives influence bacterial communities of Medicago sativa silage as determined by Illumina sequencing
Source: AMB Express. 2021 Jan 6;11:5. doi: 10.1186/s13568-020-01158-5 (PMC7788151; doi:10.1186/s13568-020-01158-5)

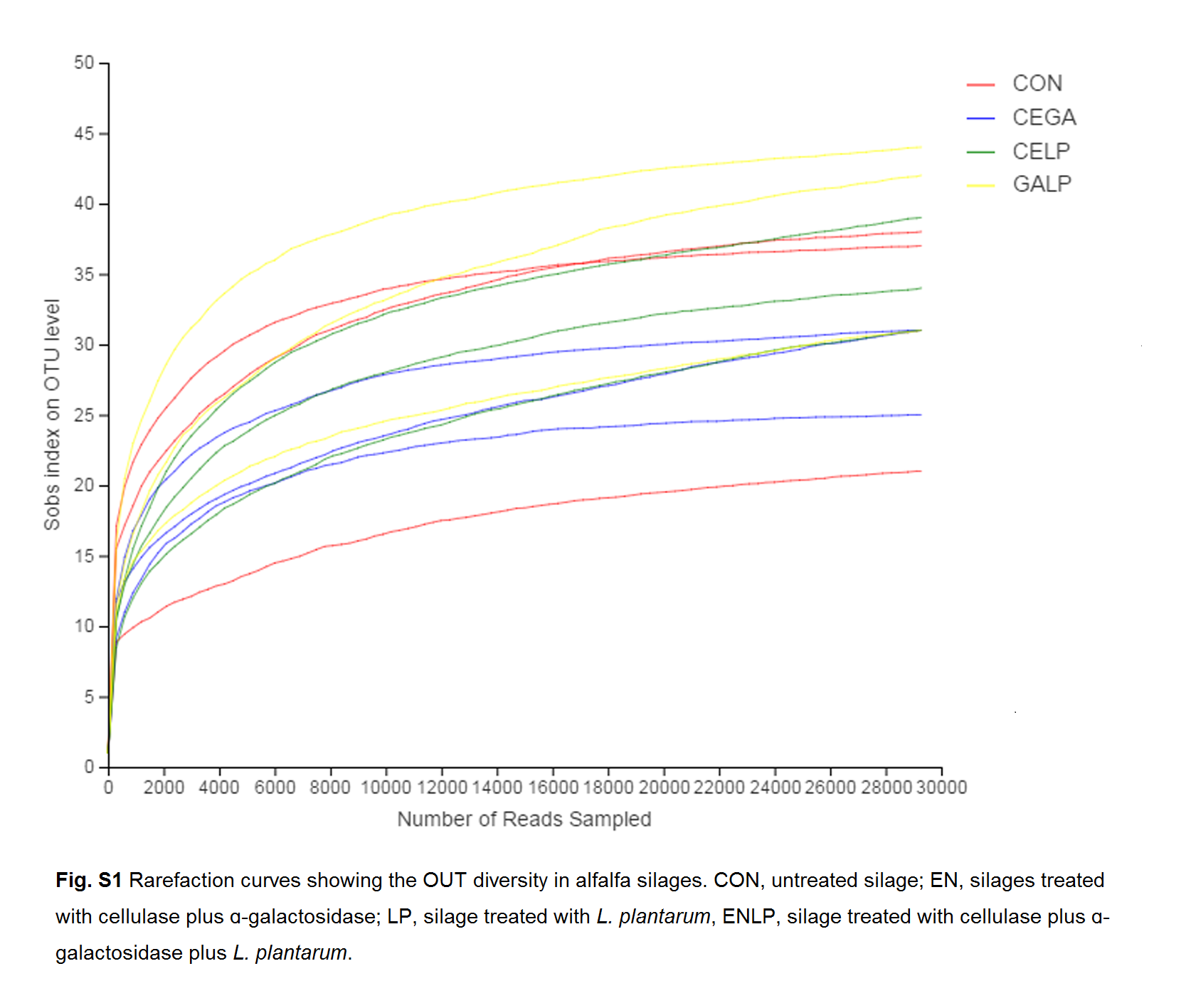

Supplement: Supplementary file 1 — Additional file 1: Figure S1. Rarefaction curves showing the OUT diversity in alfalfa silages. CON untreated silage, EN silages treated ith cellulose plus α-galactosidase, LP silage treated with L. plantarum, ENLP silage treated with cellulase plus α-galactosidase plus L. plantarum. [file 13568_2020_1158_MOESM1_ESM.png]

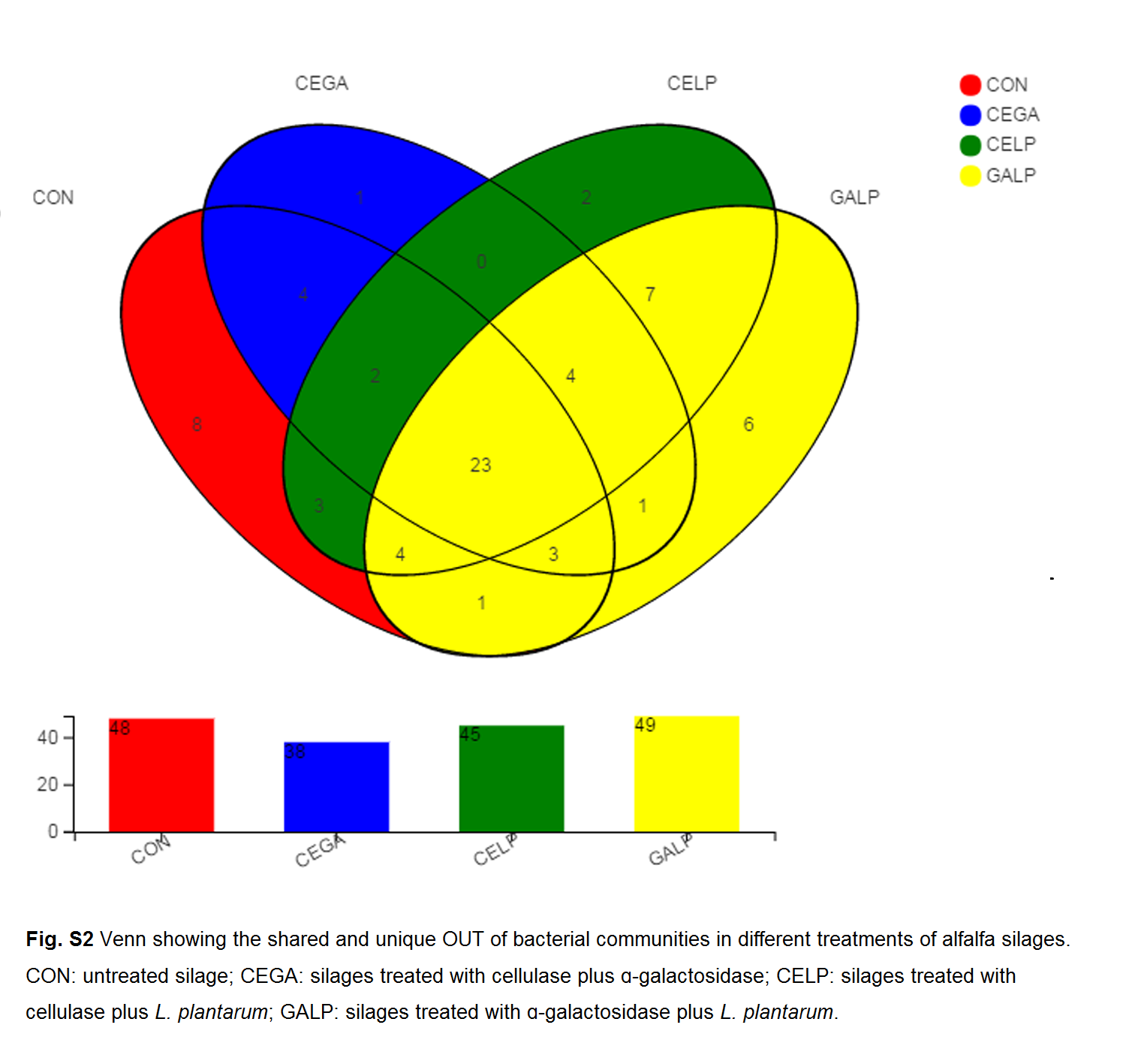

Supplement: Supplementary file 2 — Additional file 2: Figure S2. Venn showing the shared and unique OUT of bacterial communities in different treatments of alfalfa silage. CON untreated silage, CEGA silages treated with cellulase plus α-galactosidase, CELP silages treated with cellulase plus L. plantarum, GALP silages treated with α-galactosidase plus L. plantarum. [file 13568_2020_1158_MOESM2_ESM.png]

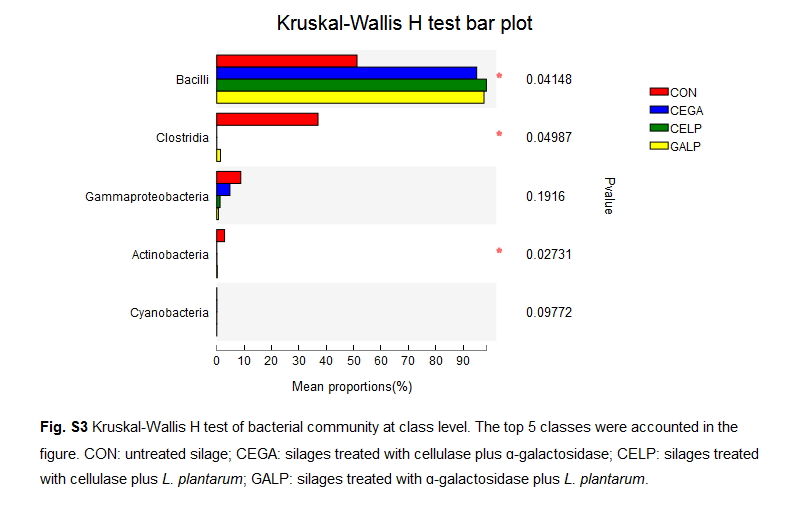

Supplement: Supplementary file 3 — Additional file 3: Figure S3. Kruskal-Wallis H test of bacterial community at class level. The top 5 classes were accounted in the figure. CON untreated silage, CEGA silages treated with cellulase plus α-galactosidase, CELP silages treated with cellulase plus L. plantarum, GALP silages treated with α-galactosidase plus L. plantarum. [file 13568_2020_1158_MOESM3_ESM.png]

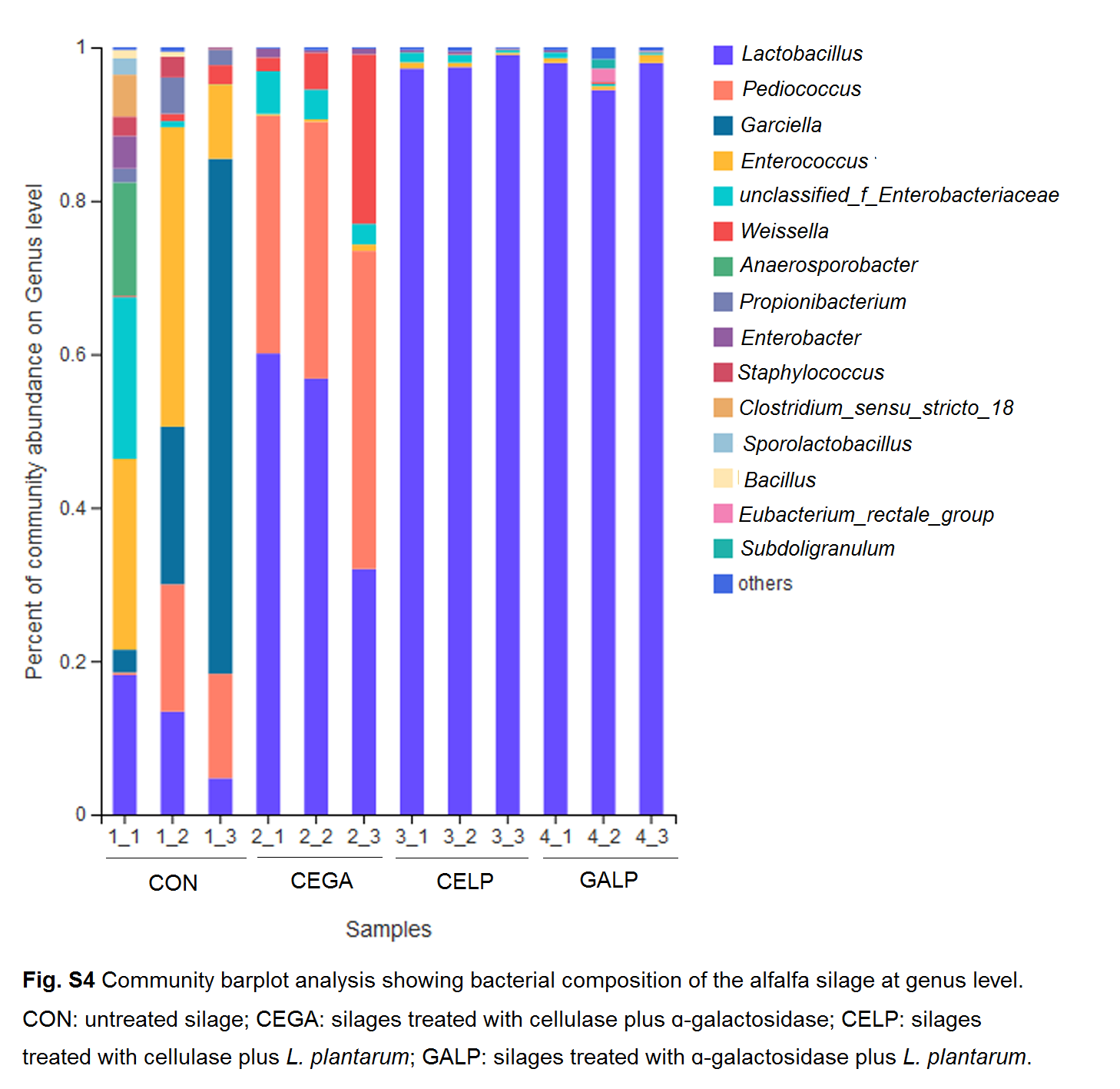

Supplement: Supplementary file 4 — Additional file 4: Figure S4. Community barplot analysis showing bacterial composition of the alfalfa silage at genus level. CON untreated silage, CEGA silages treated with cellulase plus α-galactosidase, CELP silages treated with cellulase plus L. plantarum, GALP silages treated with α-galactosidase plus L. plantarum. [file 13568_2020_1158_MOESM4_ESM.png]

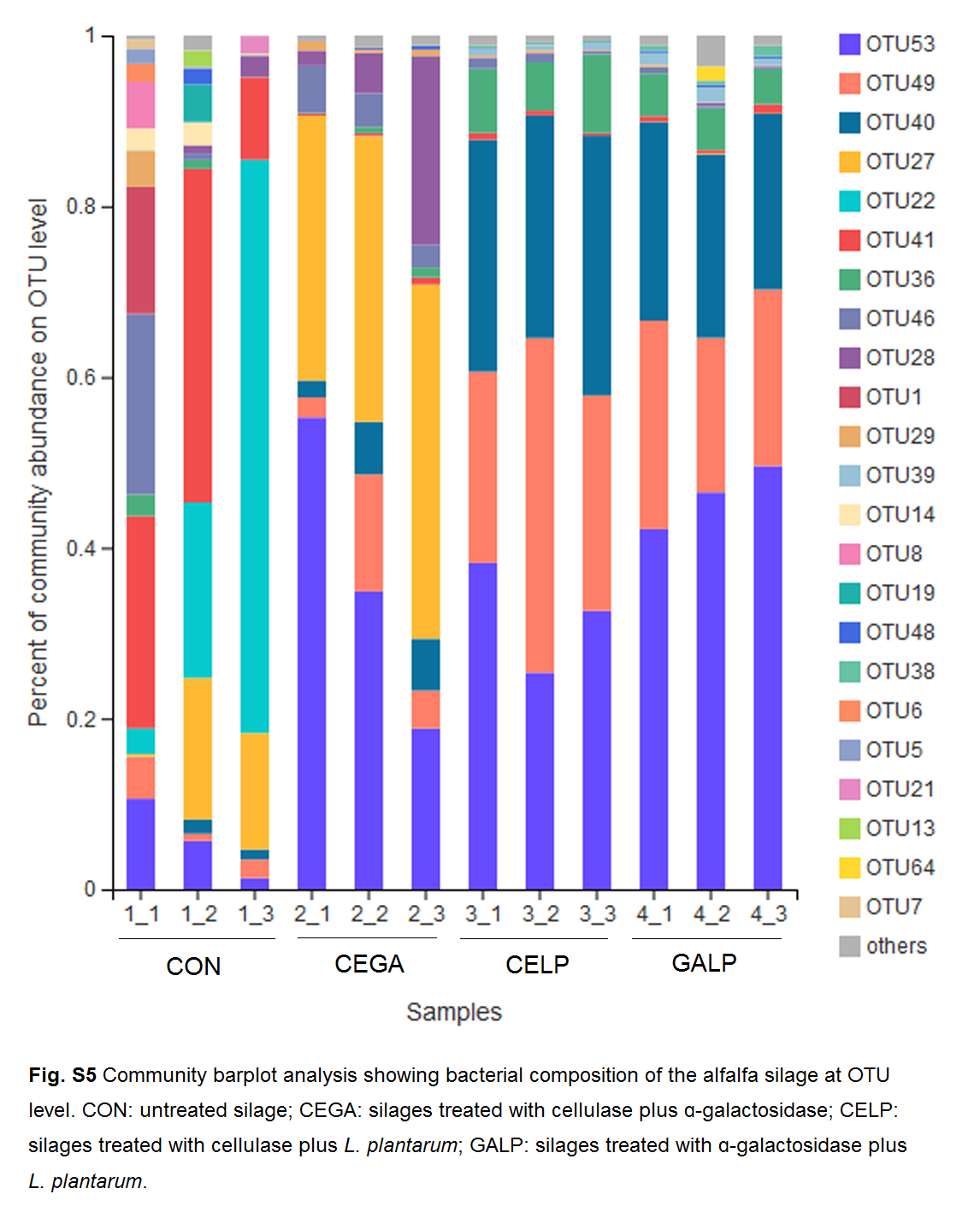

Supplement: Supplementary file 5 — Additional file 5: Figure S5. Community barplot analysis showing bacterial composition of the alfalfa silage at OUT level. CON untreated silage, CEGA silages treated with cellulase plus α-galactosidase, CELP silages treated with cellulase plus L. plantarum, GALP silages treated with α-galactosidase plus L. plantarum. [file 13568_2020_1158_MOESM5_ESM.png]
